# Mean-field analysis of synaptic alterations underlying deficient cortical gamma oscillations in schizophrenia

**DOI:** 10.21203/rs.3.rs-3938805/v1

**Published:** 2024-02-14

**Authors:** Deying Song, Daniel W. Chung, G. Bard Ermentrout

**Affiliations:** 1 Joint Program in Neural Computation and Machine Learning, Neuroscience Institute, and Machine Learning Department, Carnegie Mellon University, Pittsburgh, PA, USA, 15213.; 2 Center for the Neural Basis of Cognition, University of Pittsburgh, Pittsburgh, PA, USA, 15213.; 3 Translational Neuroscience Program, Department of Psychiatry, University of Pittsburgh, Pittsburgh, PA, USA, 15213.; 4 Department of Mathematics, University of Pittsburgh, Pittsburgh, PA, USA, 15213.

**Keywords:** mean-field model, gamma oscillations, cortical circuits, schizophrenia

## Abstract

Deficient gamma oscillations in the prefrontal cortex (PFC) of individuals with schizophrenia (SZ) are proposed to arise from alterations in the excitatory drive to fast-spiking interneurons (E→I) and in the inhibitory drive from these interneurons to excitatory neurons (I→E). Consistent with this idea, prior postmortem studies showed lower levels of molecular and structural markers for the strength of E→I and I→E synapses and also greater variability in E→I synaptic strength in PFC of SZ. Moreover, simulating these alterations in a network of quadratic integrate-and-fire (QIF) neurons revealed a synergistic effect of their interactions on reducing gamma power. In this study, we aimed to investigate the dynamical nature of this synergistic interaction at macroscopic level by deriving a mean-field description of the QIF model network that consists of all-to-all connected excitatory neurons and fast-spiking interneurons. Through a series of numerical simulations and bifurcation analyses, findings from our mean-field model showed that the macroscopic dynamics of gamma oscillations are synergistically disrupted by the interactions among lower strength of E→I and I→E synapses and greater variability in E→I synaptic strength. Furthermore, the two-dimensional bifurcation analyses showed that this synergistic interaction is primarily driven by the shift in Hopf bifurcation due to lower E→I synaptic strength. Together, these simulations predict the nature of dynamical mechanisms by which multiple synaptic alterations interact to robustly reduce PFC gamma power in SZ, and highlight the utility of mean-field model to study macroscopic neural dynamics and their alterations in the illness.

## 1 Introduction

Deficits in certain cognitive processes, such as working memory, are among the core clinical features of schizophrenia (SZ) (Kahn and Keefe, 2013; McCutcheon et al., 2020). Working memory is associated with neural population activity oscillating at gamma frequency (30–100 hertz) in the prefrontal cortex (PFC) (Fries, 2009; Miller et al., 2018), and individuals with SZ exhibit lower PFC gamma power during working memory tasks (Chen et al., 2014; Cho et al., 2006; Minzenberg et al., 2010). PFC gamma oscillations are generated, in part, by a local circuit formed by regular-spiking excitatory neurons (RSEs) and fast-spiking GABAergic interneurons (FSIs) (Gonzalez-Burgos et al., 2015). In this circuit, recurrently connected local RSEs provide excitatory drive to FSIs (E→I), and FSIs in turn provide inhibitory drive back to RSEs (I→E). Following decay of phasic inhibition from FSIs, local RSEs fire synchronously to recruit FSIs, and the repeated cycles of this reciprocal synaptic activity are thought to produce gamma oscillations in the PFC (Whittington et al., 2011, 2000). Thus, identifying neural substrates underlying lower PFC gamma power in SZ requires understanding the mechanisms by which E→I and I→E synapses regulate the generation of gamma oscillations.

Computational modeling of neural circuits has proven to be an invaluable tool for understanding dynamical mechanisms by which changes in synaptic parameters regulate the generation of gamma oscillations (Ermentrout and Chow, 2002; Jadi et al., 2016; Pittman-Polletta et al., 2015). For example, computational studies that utilized model networks of quadratic integrate-and-fire (QIF) neurons showed that key determinants of optimal gamma power include the strength of E→I and I→E synapses (Chung et al., 2022; Gonzalez-Burgos et al., 2015; Jadi and Sejnowski, 2014; Kirli et al., 2014; Rotaru et al., 2011; Spencer, 2009). In line with these predictions, prior postmortem human brain studies showed lower levels of molecular and structural markers for the strength of E→I (Chung et al., 2016a,b, 2018) and I→E (Chung et al., 2023 in press; Glausier and Lewis, 2011; Fish et al., 2021; Hashimoto et al., 2003) synapses in PFC of SZ, suggesting lower strength of these synapses in the illness. In addition, a recent study reported higher variance in the expression levels of protein markers for E→I synaptic strength across individual FSIs in PFC of SZ, and that increasing cell-to-cell variability in E→I synaptic strength disrupts gamma oscillations in a QIF model network (Chung et al., 2022). Furthermore, this study showed that SZ-associated synaptic alterations (i.e., lower E→I and I→E synaptic strength and greater variability in E→I synaptic strength) non-linearly interact to synergistically (i.e., greater than the sum of individual effect) reduce gamma power. Together, these findings provide computational insights into how small changes in multiple synaptic parameters may result in robust deficits in PFC gamma power in SZ. However, dynamical mechanisms underlying the synergistic interactions of these synaptic alterations in PFC of SZ still remain elusive.

Recent studies showed that Ott-Antonsen ansatz (Ott and Antonsen, 2008) allows a derivation of mean-field description of QIF neurons in terms of their mean firing rate and mean membrane potential (Byrne et al., 2022; Devalle et al., 2017; Gast et al., 2020; Montbrió et al., 2015; Schmidt et al., 2018). These measures can then be mathematically examined by computing bifurcation diagrams, providing an analytical tool to systematically investigate how multiple synaptic parameters interact to regulate macroscopic dynamics of neural network (Bick et al., 2020). In this study, we utilized a mean-field model of QIF neurons to investigate the dynamical mechanisms by which SZ-associated synaptic alterations interact to synergistically reduce PFC gamma power. First, we derived a mean-field description of QIF model network that consists of all-to-all connected RSEs and FSIs via AMPA, NMDA and GABA synapses. We then validated our mean-field model by comparing its gamma oscillation dynamics to that of the QIF model network across a broad spectrum of synaptic parameter choices. Finally, we performed a series of numerical simulations and two-dimensional (2D) bifurcation analyses to investigate how multiple synaptic alterations between RSEs and FSIs found in SZ may interact to non-linearly affect the generation of gamma oscillations.

## 2 Materials and Methods

### 2.1 Description of a model network of spiking neurons

Our prior studies utilized a pyramidal interneuron gamma (PING) model network that generates robust oscillatory activity at gamma frequency to simulate how gamma power is regulated by various properties of excitatory and inhibitory synapses onto regular-spiking excitatory (RSEs) and fast-spiking inhibitory (FSIs) neurons (Rotaru et al., 2011; Gonzalez-Burgos et al., 2015; Chung et al., 2022, 2023 in press). In this study, we sought to derive a set of exact equations that describe the macroscopic dynamics of a PING model network. First, we consider a PING model network that consists of RSE and FSI quadratic integrate-and-fire (QIF) point neurons. The RSEs and FSIs are reciprocally connected in an all-to-all manner via AMPA, NMDA and GABA synapses.

The membrane potential, V(t), of each neuron is governed by the following QIF model:

(1) $$C\frac{dV}{dt}=g_{l}\frac{\left(V-V_{l}\right)\left(V-V_{T}\right)}{V_{T}-V_{l}}+I_{\text{appl }}-I_{\text{syn }}\;\text{ if }V\left(t\right)\geq V_{\text{spike }},\text{ then }V\left(t\right)\leftarrow V_{R}$$

where C is the membrane capacitance, $g_{l}$ is the leak conductance, $V_{l}$ is the leak equilibrium potential, and $V_{T}$ is the threshold potential. In this model, neurons fire when V(t) reaches the spike voltage $\left(V_{\text{spike}}\right)$. Once neurons spike, their V(t) returns to the reset voltage $\left(V_{R}\right)$. Network activity is initiated by external tonic current $\left(I_{\text{appl}}\right)$ that is applied to RSEs only.

The synaptic currents $\left(I_{\text{syn}}\right)$ are the sum of AMPA $\left(g_{e}\right)$, NMDA $\left(g_{n}\right)$ and GABA $\left(g_{i}\right)$ synaptic conductance as described by the following equation:

$$I_{\text{syn}}=\left(g_{e}s_{e}+g_{n}s_{n}\right)\left(V-V_{\text{ex}}\right)+g_{i}s_{i}\left(V-V_{\text{in}}\right)$$

where $V_{\text{ex}}$ is the AMPA and NMDA equilibrium potential and $V_{\text{in}}$ is the GABA equilibrium potential. The dynamics of synaptic gating (the fraction of open channels) for AMPA $\left(s_{e}\right)$, NMDA $\left(s_{n}\right)$ and GABA $\left(s_{i}\right)$ synapses are described by the following equations:

$$\frac{ds_{e}}{dt}=-\frac{s_{e}}{\tau_{e}}+\frac{1}{N}\underset{j,k}{\sum }\delta \left(t-t_{j}^{k}\right)\;\frac{ds_{n}}{dt}=a_{n}s_{e}\left(1-s_{n}\right)-\frac{s_{n}}{\tau_{n}}\;\frac{ds_{i}}{dt}=-\frac{s_{i}}{\tau_{i}}+\frac{1}{N}\underset{j,k}{\sum }\delta \left(t-t_{j}^{k}\right)$$

where $t_{j}^{k}$ is the kth spike of the jth presynaptic neuron and N is the total number of neurons. $\tau_{e},\;\tau_{n}$ and $\tau_{i}$ are the decay time constants for AMPA, NMDA, and GABA synaptic conductance, respectively. We do not include the postsynaptic voltage effects on the NMDA channels.

### 2.2 Macroscopic description for the model network of spiking neurons

Following (Montbrió et al., 2015), we derive a set of equations that exactly describe the macroscopic dynamics of a network of all-to-all connected QIF neurons. First, we introduce a non-dimensionalized variable u, where

$$V=\frac{V_{T}+V_{l}}{2}+\frac{V_{T}-V_{l}}{2}u$$

Hence, equation (1) becomes

(2) $$C\frac{V_{T}-V_{l}}{2}\frac{du}{dt}\;=g_{l}\frac{V_{T}-V_{l}}{4}\left(u^{2}-1\right)\;-\left(g_{e}s_{e}+g_{n}s_{n}\right)\left(\frac{V_{T}+V_{l}}{2}+\frac{V_{T}-V_{l}}{2}u-V_{\text{ex}}\right)\;-g_{i}s_{i}\left(\frac{V_{T}+V_{l}+V_{l}}{2}+\frac{V_{T}-V_{l}}{2}u-V_{\text{in}}\right)+I_{\text{appl}}$$

To rewrite equation (2), we introduce the following parameters:

$${\hat{V}}_{\text{ex}}=\frac{2}{V_{T}-V_{l}}\left(V_{\text{ex}}-\frac{V_{T}+V_{l}}{2}\right)\;{\hat{V}}_{\text{in}}=\frac{2}{V_{T}-V_{l}}\left(V_{\text{in}}-\frac{V_{T}+V_{l}}{2}\right)\;\hat{I}=\frac{2I_{\text{appl}}}{g_{l}\left(V_{T}-V_{l}\right)}\;\tau =\frac{C}{g_{l}}\;{\hat{g}}_{e}=\frac{g_{e}}{g_{l}}\;{\hat{g}}_{n}=\frac{g_{n}}{g_{l}}\;{\hat{g}}_{i}=\frac{g_{i}}{g_{l}}$$

Substituting these parameters into equation (2), we have:

(3) $$\tau \frac{du}{dt}=\frac{1}{2}\left(u^{2}-1\right)-\left({\hat{g}}_{e}s_{e}+{\hat{g}}_{n}s_{n}\right)\left(u-{\hat{V}}_{\text{ex}}\right)\;-{\hat{g}}_{i}s_{i}\left(u-{\hat{V}}_{\text{in}}\right)+\hat{I}$$

Then, we rewrite equation (3) in a form of the following equation:

(4) $$\frac{du}{dt}=Au^{2}+Bu+C$$

where

$$A=\frac{1}{2\tau }\;B=-\frac{1}{\tau }\left({\hat{g}}_{e}s_{e}+{\hat{g}}_{n}s_{n}+{\hat{g}}_{i}s_{i}\right)\;C=\frac{1}{\tau }\left[\left({\hat{g}}_{e}s_{e}+{\hat{g}}_{n}s_{n}\right){\hat{V}}_{\text{ex}}+{\hat{g}}_{i}s_{i}{\hat{V}}_{\text{in}}+\hat{I}-\frac{1}{2}\right]$$

Here, we assume that $g_{e},\;g_{n},\;g_{i}$ and $I_{\text{appl}}$ conform to a Lorentzian distribution with the mode of ${\overset{‾}{g}}_{e},\;{\overset{‾}{g}}_{n},\;{\overset{‾}{g}}_{i}$ and $\overset{‾}{I}$, and the half-width of $\gamma_{e},\;\gamma_{n},\;\gamma_{i}$ and Δ, respectively. Subsequently, $g_{e},\;g_{n},\;g_{i}$ and $I_{\text{appl}}$ can be expressed by the following equations:

$$g_{e}={\bar{g}}_{e}+\gamma_{e}\xi_{e}\;g_{n}={\bar{g}}_{n}+\gamma_{n}\xi_{n}\;g_{i}={\bar{g}}_{i}+\gamma_{i}\xi_{i}\;I_{\text{appl}}=\bar{I}+\Delta \eta$$

where $\xi_{e},\;\xi_{n},\;\xi_{i}$ and η have the Lorentzian distribution, $p(\xi )=1/\left[\pi \left(1+\xi^{2}\right)\right]$.

We now let the size of the network go to infinity and set $V_{\text{spike}}=+\infty$ and $V_{R}=-\infty$, so that our QIF model is equivalent to a theta model and we can employ the methods of (Montbrió et al., 2015). Given the conservation of number of neurons in the network, we have the following continuity equation for the probability density function $\rho \left(u|\xi_{e},\xi_{n},\xi_{i},\eta ,t\right)$:

(5) $$\frac{\partial \rho }{\partial t}+\frac{\partial }{\partial u}\left[\rho \left(Au^{2}+Bu+C\right)\right]=0$$

where $\rho \left(Au^{2}+Bu+C\right)$ is the flux of membrane potentials of QIF neurons. Based on (Montbrió et al., 2015), we assume that the solution of equation (5) converges to a Lorentzian-shape function independent of the initial conditions. This ansatz is expressed by the following equation:

(6) $$\rho \left(u|\xi_{e},\xi_{n},\xi_{i},\eta ,t\right)=\frac{1}{\pi }\frac{\alpha \left(\xi_{e},\xi_{n},\xi_{i},\eta ,t\right)}{{\left[u-\beta \left(\xi_{e},\xi_{n},\xi_{i},\eta ,t\right)\right]}^{2}+\alpha {\left(\xi_{e},\xi_{n},\xi_{i},\eta ,t\right)}^{2}}$$

where $\beta \left(\xi_{e},\xi_{n},\xi_{i},\eta ,t\right)$ and $\alpha \left(\xi_{e},\xi_{n},\xi_{i},\eta ,t\right)$ are the mode and the half-width, respectively, of the Lorentzian distribution.

We now abbreviate $\alpha \left(\xi_{e},\xi_{n},\xi_{i},\eta ,t\right)$ as α and $\beta \left(\xi_{e},\xi_{n},\xi_{i},\eta ,t\right)$ as β, and let $\alpha^{\prime }$ denote $\frac{\partial \alpha }{\partial t}$ and $\beta^{\prime }$ denote $\frac{\partial \beta }{\partial t}$. Substituting the ansatz into the continuity equation and equating powers of u, we find that:

$$\alpha^{\prime }=2A\beta \alpha +B\alpha \;\beta^{\prime }=A\left(\beta^{2}-\alpha^{2}\right)+C+B\beta$$

Letting, w=β+iα, we find

(7) $$\frac{dw}{dt}=Aw^{2}+Bw+C$$

The firing rate of a neuron with parameters $\Xi =\left(\xi_{i},\xi_{n},\xi_{i},\eta \right)\in R^{4}$ is the flux at infinity:

$$r\left(\Xi ,t\right)=\underset{u\to \infty }{\text{lim}}\;\rho \left(u,\Xi ,t\right)\left(Au^{2}+Bu+C\right)=\frac{A\alpha \left(\Xi ,t\right)}{\pi }$$

and the mean firing rate of the population of neurons is

(8) $$r\left(t\right)=\int_{{\mathbb{R}}^{4}}P\left(\Xi \right)r\left(\Xi ,t\right)d\Xi$$

where P(Ξ) is the joint probability density for the four Lorentzian distributed parameters, Ξ.

The dynamics of synaptic gating for AMPA synapses are rewritten as:

$$\frac{ds_{e}}{dt}=\frac{1}{N}\overset{N}{\underset{j=1}{\sum }}\frac{ds_{e,j}}{dt}\;=\frac{1}{N}\overset{N}{\underset{j=1}{\sum }}\left(-\frac{s_{e,j}}{\tau_{e}}+\frac{1}{N}\underset{i,k}{\sum }\delta \left(t-t_{i}^{k}\right)\right)\;=-\frac{s_{e}}{\tau_{e}}+r_{e}$$

Similarly, the dynamics of synaptic gating for GABA synapses are rewritten as:

$$\frac{ds_{i}}{dt}=-\frac{s_{i}}{\tau_{i}}+r_{i}$$

As for the dynamics of synaptic gating for NMDA synapses,

$$\frac{ds_{n}}{dt}=\frac{1}{N}\underset{j}{\sum }\frac{ds_{n,j}}{dt}\;=\frac{1}{N}\underset{j}{\sum }a_{n}s_{e,j}\left(1-s_{n,j}\right)-\frac{s_{n}}{\tau_{n}}\;\approx a_{n}\left(\frac{1}{N}\underset{j}{\sum }s_{e,j}\right)\left(1-\frac{1}{N}\underset{j}{\sum }s_{n,j}\right)-\frac{s_{n}}{\tau_{n}}$$

So that

$$\frac{ds_{n}}{dt}=a_{n}s_{e}\left(1-s_{n}\right)-\frac{s_{n}}{\tau_{n}}$$

In order to obtain a closed system for the mean firing rate, mean potential, and synaptic gating variables, we need to evaluate the integral in equation (8). Since $\xi_{e},\;\xi_{n},\;\xi_{i}$ and η are Lorentzian distributed, Cauchy’s residue theorem is used to perform integration as previously described (Montbrió et al., 2015), yielding:

(9) $$r\left(t\right)=\frac{\alpha \left(\sigma_{e}i,\sigma_{n}i,\sigma_{i}i,\sigma i,t\right)}{2\pi \tau }$$

where $\sigma_{e},\;\sigma_{n},\;\sigma_{i},\;\sigma \in \{-1,+1\}$ are to be determined by the signs of the terms in the ODEs, so that the firing rate does not fall below zero.

Similarly, the mean membrane potential of QIF neurons, β, with $\xi_{e},\;\xi_{n},\;\xi_{i}$, and η at time t is expressed by the following equation:

$$\beta \left(\xi_{e},\xi_{n},\xi_{i},\eta ,t\right)=\text{p}.\text{v}.\;{\int^{\text{​}}}_{-\infty }^{\infty }\rho \left(u|\xi_{e},\xi_{n},\xi_{i},\eta ,t\right)u\;du$$

As $\xi_{e},\;\xi_{n},\;\xi_{i}$ and η are Lorentzian distributed, Cauchy’s residue theorem is used to obtain the population membrane potential, u, so that

(10) $$u\left(t\right)=\beta \left(\sigma_{e}i,\sigma_{n}i,\sigma_{i}i,\sigma i,t\right)$$

where $\sigma_{e},\;\sigma_{n},\;\sigma_{i}$ and σ take the same values as those in equation (9). Substituting $g_{e}={\overset{‾}{g}}_{e}+\gamma_{e}\sigma_{e}i,\;g_{n}={\overset{‾}{g}}_{n}+\gamma_{n}\sigma_{n}i,\;g_{i}={\overset{‾}{g}}_{i}+\gamma_{i}\sigma_{i}i$, and $I=\overset{‾}{I}+\Delta \sigma i$ into equation (7), we have

$$Aw^{2}=\frac{1}{2\tau }\left(\beta^{2}-\alpha^{2}+2\alpha \beta i\right)\;Bw=-(\frac{\left({\bar{g}}_{e}+\gamma_{e}\sigma_{e}i+{\bar{g}}_{n}+\gamma_{n}\sigma_{n}i\right)s_{e}}{\tau g_{l}}\;+\frac{\left({\bar{g}}_{i}+\gamma_{i}\sigma_{i}i\right)s_{i}}{\tau g_{l}})\left(\beta +\alpha i\right)\;C=\frac{\left({\bar{g}}_{e}+\gamma_{e}\sigma_{e}i+{\bar{g}}_{n}+\gamma_{n}\sigma_{n}i\right)s_{e}}{\tau g_{l}}{\hat{V}}_{\text{ex}}\;+\frac{\left({\bar{g}}_{i}+\gamma_{i}\sigma_{i}i\right)s_{i}}{\tau g_{l}}{\hat{V}}_{\text{in}}\;+\frac{2}{g_{l}\left(V_{T}-V_{l}\right)\tau }\left(\bar{I}+i\Delta \sigma \right)-\frac{1}{2\tau }$$

Thus,

$$\frac{d\alpha }{dt}=\text{Im}\left(\frac{dw}{dt}\right)\;=\frac{\alpha \beta }{\tau }-\left(\frac{{\bar{g}}_{e}s_{e}+{\bar{g}}_{n}s_{n}+{\bar{g}}_{i}s_{i}}{\tau g_{l}}\right)\alpha \;-\frac{\left(\gamma_{e}\sigma_{e}+\gamma_{n}\sigma_{n}\right)s_{e}}{\tau g_{l}}\left(\beta -{\hat{V}}_{\text{ex}}\right)\;-\frac{\gamma_{i}s_{i}\sigma_{i}}{\tau g_{l}}\left(\beta -{\hat{V}}_{\text{in}}\right)+\frac{2\Delta \sigma }{g_{l}\left(V_{T}-V_{l}\right)\tau }$$

We must guarantee that the mean firing rate, r=α/π, is never negative and that $\frac{d\alpha }{dt}>0$ when α=0, which implies that:

$$\sigma_{e}=\text{sgn}\left({\hat{V}}_{\text{ex }}-\beta \right)=\text{sgn}\left({\hat{V}}_{\text{ex }}-u\right)\;\sigma_{n}=\text{sgn}\left({\hat{V}}_{\text{ex }}-\beta \right)=\text{sgn}\left({\hat{V}}_{\text{ex }}-u\right)\;\sigma_{i}=\text{sgn}\left({\hat{V}}_{\text{in }}-\beta \right)=\text{sgn}\left({\hat{V}}_{\text{in }}-u\right)\;\sigma =1$$

Therefore, ODEs for the population firing rate and membrane potential become, respectively,

$$\frac{dr}{dt}=\frac{ru}{\tau }-\left(\frac{{\bar{g}}_{e}s_{e}+{\bar{g}}_{n}s_{n}+{\bar{g}}_{i}s_{i}}{\tau g_{l}}\right)r\;+\frac{1}{2\pi \tau }(\frac{2\Delta }{g_{l}\left(V_{T}-V_{l}\right)\tau }\;+\frac{\left(\gamma_{e}+\gamma_{n}\right)s_{e}}{\tau g_{l}}\left|u-{\hat{V}}_{\text{ex}}\right|+\frac{\gamma_{i}s_{i}}{\tau g_{l}}\left|u-{\hat{V}}_{\text{in}}\right|)$$

and

$$\frac{du}{dt}=\frac{u^{2}}{2\tau }-2\tau \pi^{2}r^{2}\;+\frac{\left({\bar{g}}_{e}+{\bar{g}}_{n}\right)s_{e}}{\tau g_{l}}\left(u-{\hat{V}}_{\text{ex}}\right)-\frac{g_{i}s_{i}}{\tau g_{l}}\left(u-{\hat{V}}_{\text{in}}\right)\;+2\pi r(\frac{\left(\gamma_{e}+\gamma_{n}\right)s_{e}}{g_{l}}\text{sgn}\left({\hat{V}}_{\text{ex}}-u\right)\;+\frac{\gamma_{i}s_{i}}{g_{l}}\text{sgn}\left({\hat{V}}_{\text{in}}-u\right))\;+\frac{2\bar{I}}{g_{l}\left(V_{T}-V_{l}\right)\tau }-\frac{1}{2\tau }$$

Since the PING network consists of all-to-all connected RSEs and FSIs, the ODEs for the population firing rate and membrane potentials can be expressed as below to construct a mean-field model of PING network; the parameters for RSEs and FSIs are denoted with (e) and (i), respectively:

(11) $$\frac{dr^{\left(e\right)}}{dt}=\frac{r^{\left(e\right)}u^{\left(e\right)}}{\tau }-\left(\frac{{\bar{g}}_{ee}s^{\left(e\right)}}{\tau g_{l}}+\frac{{\bar{g}}_{ei}s^{\left(i\right)}}{\tau g_{l}}+\frac{{\bar{g}}_{en}s^{\left(n\right)}}{\tau g_{l}}\right)r^{\left(e\right)}\;+\frac{1}{2\pi \tau }(\frac{2\Delta_{e}}{g_{l}\left(V_{T}-V_{l}\right)\tau }+\frac{\gamma_{ee}s^{\left(e\right)}}{\tau g_{l}}\left|u^{\left(e\right)}-{\hat{V}}_{\text{ex}}\right|\;+\frac{\gamma_{ei}s^{\left(i\right)}}{\tau g_{l}}\left|u^{\left(e\right)}-{\hat{V}}_{\text{in}}\right|+\frac{\gamma_{en}s^{\left(n\right)}}{\tau g_{l}}\left|u^{\left(e\right)}-{\hat{V}}_{\text{ex}}\right|)\;\frac{du^{\left(e\right)}}{dt}=\frac{u^{{\left(e\right)}^{2}}}{2\tau }-2\pi^{2}\tau r^{{\left(e\right)}^{2}}-\frac{{\bar{g}}_{ee}s^{\left(e\right)}}{\tau g_{l}}\left(u^{\left(e\right)}-{\hat{V}}_{\text{ex}}\right)\;-\frac{{\bar{g}}_{ei}s^{\left(i\right)}}{\tau g_{l}}\left(u^{\left(e\right)}-{\hat{V}}_{\text{in}}\right)-\frac{{\bar{g}}_{en}s^{\left(n\right)}}{\tau g_{l}}\left(u^{\left(e\right)}-{\hat{V}}_{\text{ex}}\right)\;+2\pi r^{\left(e\right)}(\frac{\gamma_{ee}s^{\left(e\right)}}{g_{l}}\text{sgn}\left({\hat{V}}_{\text{ex}}-u^{\left(e\right)}\right)\;+\frac{\gamma_{ei}s^{\left(i\right)}}{g_{l}}\text{sgn}\left({\hat{V}}_{\text{in}}-u^{\left(e\right)}\right)\;+\frac{\gamma_{en}s^{\left(n\right)}}{g_{l}}\text{sgn}\left({\hat{V}}_{\text{ex}}-u^{\left(e\right)}\right))\;\;+\frac{2{\bar{I}}_{e}}{g_{l}\left(V_{T}-V_{l}\right)\tau }-\frac{1}{2\tau }\;\frac{ds^{\left(e\right)}}{dt}=-\frac{s^{\left(e\right)}}{\tau_{e}}+r^{\left(e\right)}\;\frac{dr^{\left(i\right)}}{dt}=\frac{r^{\left(i\right)}u^{\left(i\right)}}{\tau }-\left(\frac{{\bar{g}}_{ie}s^{\left(e\right)}}{\tau g_{l}}+\frac{{\bar{g}}_{ii}s^{\left(i\right)}}{\tau g_{l}}+\frac{{\bar{g}}_{\text{in }}s^{\left(n\right)}}{\tau g_{l}}\right)r^{\left(i\right)}\;+\frac{1}{2\pi \tau }(\frac{2\Delta_{i}}{g_{l}\left(V_{T}-V_{l}\right)\tau }+\frac{\gamma_{ie}s^{\left(e\right)}}{\tau g_{l}}\left|u^{\left(i\right)}-{\hat{V}}_{\text{ex}}\right|\;+\frac{\gamma_{ii}s^{\left(i\right)}}{\tau g_{l}}\left|u^{\left(i\right)}-{\hat{V}}_{\text{in}}\right|+\frac{\gamma_{\text{in}}s^{\left(n\right)}}{\tau g_{l}}\left|u^{\left(i\right)}-{\hat{V}}_{\text{ex}}\right|)\;\frac{du^{\left(i\right)}}{dt}=\frac{u^{{\left(i\right)}^{2}}}{2\tau }-2\pi^{2}\tau r^{{\left(i\right)}^{2}}-\frac{{\bar{g}}_{ie}s^{\left(e\right)}}{\tau g_{l}}\left(u^{\left(i\right)}-{\hat{V}}_{\text{ex}}\right)\;-\frac{{\bar{g}}_{ii}s^{\left(i\right)}}{\tau g_{l}}\left(u^{\left(i\right)}-{\hat{V}}_{\text{in }}\right)-\frac{{\bar{g}}_{\text{in }}s^{\left(n\right)}}{\tau g_{l}}\left(u^{\left(i\right)}-{\hat{V}}_{\text{ex }}\right)\;+2\pi r^{\left(i\right)}(\frac{\gamma_{ie}s^{\left(e\right)}}{g_{l}}\text{sgn}\left({\hat{V}}_{\text{ex}}-u^{\left(i\right)}\right)\;+\frac{\gamma_{ii}s^{\left(i\right)}}{g_{l}}\text{sgn}\left({\hat{V}}_{\text{in}}-u^{\left(i\right)}\right)\;+\frac{\gamma_{\text{in}}s^{\left(n\right)}}{g_{l}}\text{sgn}\left({\hat{V}}_{\text{ex}}-u^{\left(i\right)}\right))\;+\frac{2{\bar{I}}_{i}}{g_{l}\left(V_{T}-V_{l}\right)\tau }-\frac{1}{2\tau }\;\frac{ds^{\left(i\right)}}{dt}=-\frac{s^{\left(i\right)}}{\tau_{i}}+r^{\left(i\right)}\;\frac{ds^{\left(n\right)}}{dt}=a_{n}s^{\left(e\right)}\left(1-s^{\left(n\right)}\right)-\frac{s^{\left(n\right)}}{\tau_{n}}$$

## 3 Results

### 3.1 Comparing the dynamics of gamma oscillations between QIF network and mean-field model

**Fig. 1:**
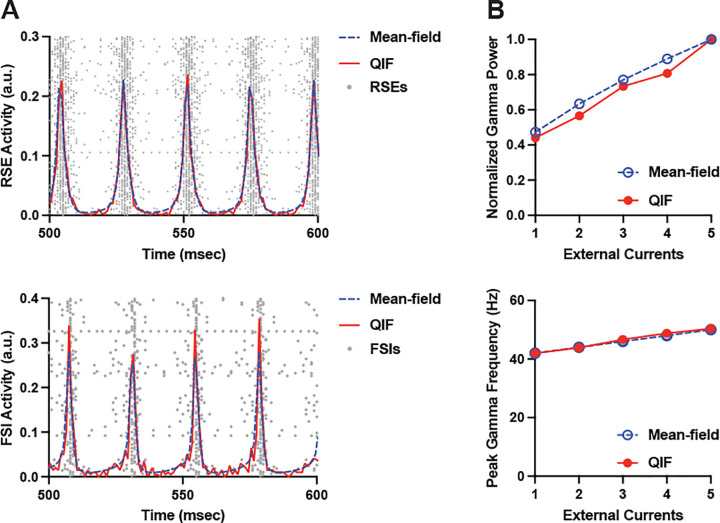
Comparing gamma oscillation dynamics between QIF model network and mean-field model. (A) Representative average spiking activity (red) and raster plots (gray) of QIF model network and the mean firing rate (blue) of mean-field model for RSEs (up) and FSIs (bottom) at $I_{\text{appl}}$ (QIF) or $\overset{‾}{I}$ (mean-field) = 2. QIF network consisted of 800 RSEs and 200 FSIs. Superimposing the mean firing rate of mean-field model to the average spiking activity of QIF network shows an overlapping gamma oscillation dynamics for both RSEs and FSIs. (B) Increasing $I_{\text{appl}}$ and $\overset{‾}{I}$ in QIF network and mean-field model, respectively, results in congruent increases in gamma power and peak gamma frequency, highlighting a close alignment between the two models. Results for the QIF model network are the average of 100 trials.

To explore how our mean-field model captures the gamma oscillation dynamics of a QIF model network composed of all-to-all connected RSEs and FSIs, we first performed a qualitative comparison of the activities of RSEs and FSIs between the two models (Figure.1A). Consistent with our prior studies, applying external currents to RSEs resulted in synchronous firings of RSEs and FSIs at gamma frequency in the QIF model network. Similarly, applying external current to RSEs in the mean-field model produced oscillatory activities of RSEs and FSIs at gamma frequency. Superimposing the firing rate of mean-field model to the spiking activity of QIF model network for both RSEs and FSIs showed overlapping gamma oscillation dynamics, demonstrating a close alignment between the two models.

To further compare gamma oscillation dynamics between the QIF network and mean-field model, we quantified changes in the gamma power and the peak gamma frequency in response to increasing external current (Figure.1B). Increasing the strength of external current to RSEs augmented gamma power to a similar extent between the QIF network and the mean-field model. Furthermore, increasing the external current led to identical increments in peak gamma frequency in both models. Together, these findings demonstrate the robustness of mean-field model in extending the gamma oscillation dynamics observed in the QIF model network to the macroscopic level.

### 3.2 Characterizing the effect of synaptic strength on gamma power in mean-field model

Multiple studies using *in vivo* models have shown that E→I synaptic strength is a critical determinant of cortical gamma power (Sohal et al., 2009; Carlen et al., 2012; Pelkey et al., 2015). To investigate how E→I synaptic strength regulates gamma oscillation dynamics in the mean-field model, we characterized the effect of mean AMPA-mediated $\left({\overset{‾}{g}}_{E\to I}\right)$ and mean NMDA-mediated $\left({\overset{‾}{g}}_{N\to I}\right)$ conductance in FSIs on gamma power.

**Fig. 2:**
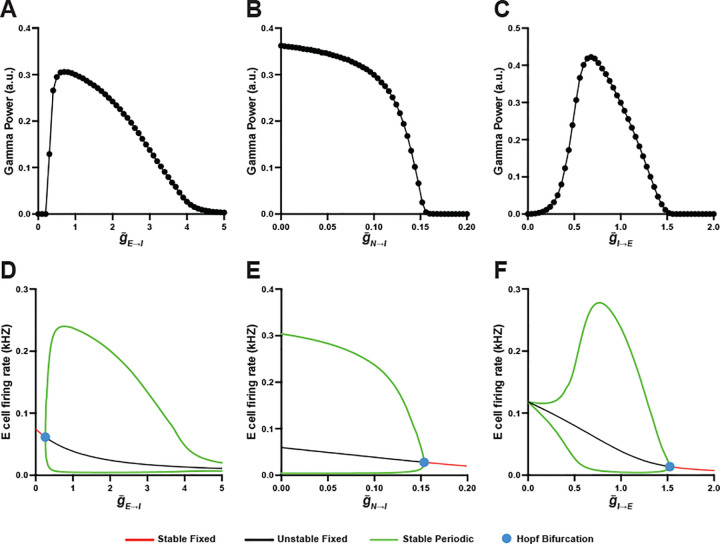
Effect of E→I and I→E synaptic strengths on gamma power in mean-field model. (A-C) Numerical simulations showing the effect of ${\overset{‾}{g}}_{E\to I}$ (A), ${\overset{‾}{g}}_{N\to I}$ (B) and ${\overset{‾}{g}}_{I\to E}$ (C) on gamma power. ${\overset{‾}{g}}_{E\to I}$ and ${\overset{‾}{g}}_{I\to E}$ form an inverted-U relationship with gamma power, whereas increasing ${\overset{‾}{g}}_{N\to I}$ progressively reduces gamma power. (D-F) Bifurcation diagrams reveal the Hopf bifurcation points (blue dots) at ${\overset{‾}{g}}_{E\to I}=0.26$ (D), ${\overset{‾}{g}}_{N\to I}=0.15$ (E) and ${\overset{‾}{g}}_{I\to E}=1.52$ (F).

First, we numerically simulated the effect of ${\overset{‾}{g}}_{E\to I}$ on gamma power (Figure.2A). Gamma power sharply increased as ${\overset{‾}{g}}_{E\to I}$ increased from zero and reached a peak at ${\overset{‾}{g}}_{E\to I}=0.7$. As ${\overset{‾}{g}}_{E\to I}$ continued to increase beyond this point, gamma power began to decline, eventually reaching zero at ${\overset{‾}{g}}_{E\to I}=5$. These changes resulted in an inverted-U relationship between ${\overset{‾}{g}}_{E\to I}$ and gamma power, which mirrors the findings from a QIF model network in our prior study (Chung et al., 2022). The inverted-U relationship was also observed in the bifurcation diagram, which showed a Hopf bifurcation point at ${\overset{‾}{g}}_{E\to I}=0.26$ marking the onset of gamma oscillations (Figure.2D).

Next, we assessed the effect of ${\overset{‾}{g}}_{N\to I}$ on gamma power (Figure.2B). Numerical simulation showed maximal gamma power at ${\overset{‾}{g}}_{N\to I}=0$, which then gradually decreased as ${\overset{‾}{g}}_{N\to I}$ was increased to 0.12. Further increasing ${\overset{‾}{g}}_{N\to I}$ resulted in a sharp decrease in gamma power, which reached zero at ${\overset{‾}{g}}_{N\to I}=0.15$. Consistent with these changes, a Hopf bifurcation point was observed at ${\overset{‾}{g}}_{N\to I}=0.15$ corresponding to the offset of gamma oscillations (Figure.2E). These findings align with a prior finding in a QIF model network in which increasing NMDA-mediated conductance in FSIs reduced gamma power (Rotaru et al., 2011).

In addition to E→I synapses, I→E synaptic strength is proposed to be another critical determinant of cortical gamma power (Cho et al., 2015; Berryer et al., 2016). To investigate the relationship between I→E synaptic strength and gamma oscillation dynamics in the mean-field model, we characterized the effect of mean GABA-mediated conductance in RSEs $\left({\overset{‾}{g}}_{I\to E}\right)$ on gamma power.

Numerical simulation showed that gamma power sharply increased as ${\overset{‾}{g}}_{I\to E}$ increased from zero and reached a peak at ${\overset{‾}{g}}_{I\to E}=0.68$ (Figure.2C). Further increasing ${\overset{‾}{g}}_{I\to E}$ resulted in a sharp decrease in gamma power which reached zero at ${\overset{‾}{g}}_{I\to E}=1.52$. In line with these findings, the bifurcation diagram showed a Hopf bifurcation point at ${\overset{‾}{g}}_{I\to E}=1.52$ corresponding to the offset of gamma oscillations (Figure.2F). Thus, similar to ${\overset{‾}{g}}_{E\to I},\;{\overset{‾}{g}}_{I\to E}$ also formed an inverted-U relationship with gamma power, consistent with its effect in a QIF model network in our prior study (Chung et al., 2022).

### 3.3 Characterizing the effect of synaptic variability on gamma power in mean-field model

Excitatory synaptic inputs to cortical neurons intrinsically vary in their strength (Ko et al., 2011; Hofer et al., 2011) and a recent study showed that increasing the variability in E→I synaptic strength monotonically reduces gamma power in a QIF model network (Chung et al., 2022). To characterize the relationship between the variability in E→I synaptic strength and gamma oscillation dynamics in the mean-field model, we assessed how gamma power changes in response to increasing the half-width of AMPA-mediated $\left(\gamma_{E\to I}\right)$ and NMDA-mediated $\left(\gamma_{N\to I}\right)$ conductance across FSIs.

**Fig. 3:**
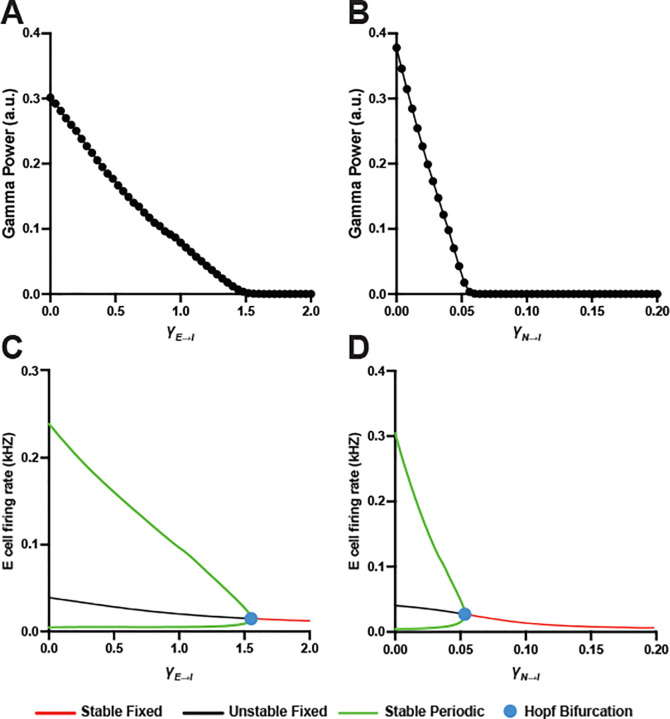
Effect of E→I synaptic variability on gamma power in mean-field model. (A-B) Numerical simulations show a progressive decline in gamma power with greater $\gamma_{E\to I}$ (A) and $\gamma_{N\to I}$ (B). (C-D) Bifurcation diagrams reveal the Hopf bifurcation points (blue dots) at $\gamma_{E\to I}=1.55$ (C) and $\gamma_{N\to I}=0.053$ (D).

Increasing $\;\gamma_{E\to I}$ progressively reduced gamma power, which reached zero at $\gamma_{E\to I}=1.55$ (Figure.3A). Consistently, the bifurcation diagram showed a Hopf bifurcation point at $\gamma_{E\to I}=1.55$, corresponding to an offset of gamma oscillations (Figure.3C). Similarly, increasing $\gamma_{N\to I}$ progressively decreased gamma power (Figure.3B); however, a Hopf bifurcation point marking an offset of gamma oscillations occurred at $\gamma_{N\to I}=0.053$ (Figure.3D), suggesting the effect of $\gamma_{N\to I}$ on gamma power is more robust than that of $\gamma_{E\to I}$.

### 3.4 Synergistic interactions of SZ-associated synaptic alterations in mean-field model

Above findings show that our mean-field model reliably captures changes in gamma oscillation dynamics in response to shifts in mean and variability of synaptic strengths at the macroscopic level. Prior postmortem human brain studies suggest that these synaptic parameters are altered in SZ (Table.1). For example, we previously reported fewer E→I synapses in PFC of SZ (Chung et al., 2016a). Also, multiple studies showed lower levels of the GABA-synthesizing enzyme glutamic acid decarboxylase 67 (GAD67), a marker for I→E synaptic strength, in PFC of SZ (Glausier and Lewis, 2011; Fish et al., 2021; Hashimoto et al., 2003). Moreover, we recently reported greater coefficient of variation in the levels of vesicular glutamate transporter 1 (VGlut1) and postsynaptic density 95 (PSD95), markers of E→I synaptic strength, within E→I synapses across FSIs in PFC of SZ (Chung et al., 2022). Finally, prior simulations in a QIF model network showed that these SZ-associated alterations (i.e., lower E→I strength, lower I→E strength and greater variability in E→I strength) interact to synergistically (greater than the sum of individual impact) reduce gamma power (Chung et al., 2022). In light of these findings, we explored if the synergistic interactions of these synaptic alterations can be captured by our mean-field model.

**Fig. 4:**
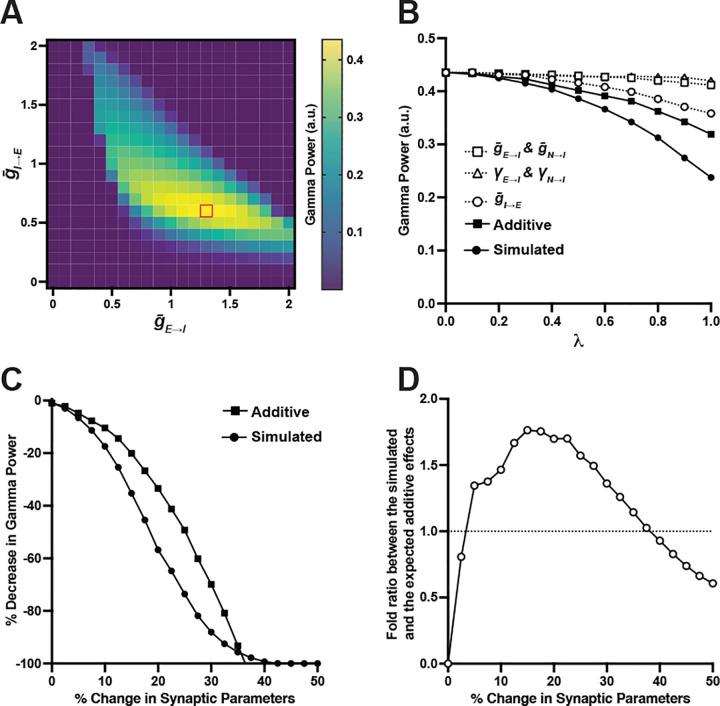
Numerical simulations of the interactions among the synaptic alterations found in PFC of SZ on gamma power. (A) Simulating the effect of interaction between E→I and I→E synaptic strengths reveals optimal gamma power (red square) at ${\overset{‾}{g}}_{E\to I}=1.3,\;\;{\overset{‾}{g}}_{N\to I}=0.13$ and ${\overset{‾}{g}}_{I\to E}=0.6$ when $\gamma_{E\to I},\;\gamma_{N\to I}$ and $\gamma_{I\to E}$ were set at 0.01; these parameter values represent the healthy state. The x-axis represents the parameter range for ${\overset{‾}{g}}_{E\to I}$, while ${\overset{‾}{g}}_{N\to I}$ was set at 0.1 of ${\overset{‾}{g}}_{E\to I}$ across each trial to maintain the fixed ratio of these two parameters. (B) Scaling λ from the healthy state (0% difference where λ=0) to SZ state (20% difference where λ=1; see Table 2 for the parameter values for these two states) for ${\overset{‾}{g}}_{E\to I}$ and ${\overset{‾}{g}}_{N\to I}$ (open square) or $\gamma_{E\to I}$ and $\gamma_{N\to I}$ (open triangle) resulted in 5% or 3% deficit in gamma power, respectively, while scaling λ for ${\overset{‾}{g}}_{I\to E}$ (open circle) led to an 18% deficits in gamma power. Simulating these changes in concert (filled circle) reduced gamma power by 45%, significantly greater than the expected 26% deficits from the additive effect (filled square), demonstrating a non-linear interaction of these synaptic parameters. (C) Comparing gamma power deficits between the expected additive effect (filled square) and the simulated effect (filled circle) of the parameter interactions across a wide range of magnitude of difference shows that the non-linear interaction occurs when the parameter difference ranges from 7.5% to 32.5%. (D) The fold ratio of gamma power deficits between the simulated effect and the additive effect shows that the non-linear interaction (marked by fold ratio > 1) is greatest when the parameter difference ranges from 12.5% to 22.5% (represented by the peak of the graph).

First, we sought to identify a set of values to represent the parameter state in healthy individuals. To establish this parameter set, we simulated the effect of interaction between ${\overset{‾}{g}}_{E\to I},\;{\overset{‾}{g}}_{N\to I}$ and ${\overset{‾}{g}}_{I\to E}$ on gamma power and searched for a set of parameter values that produced maximal gamma power (Figure.4A). In this simulation, we modeled changes in the total E→I synaptic strength by adjusting ${\overset{‾}{g}}_{E\to I}$ and ${\overset{‾}{g}}_{N\to I}$ simultaneously while maintaining the fixed ratio between ${\overset{‾}{g}}_{N\to I}$ and ${\overset{‾}{g}}_{E\to I}$ at 1:10 to reflect the NMDA-to-AMPA ratio empirically observed in FSIs (Rotaru et al., 2011). Also, we set $\gamma_{E\to I}$ and $\gamma_{N\to I}$ at 0.01, corresponding to the lower end of the range for these parameters that results in a linear decrease in gamma power (Figure.3). The parameter values that produced maximum gamma power occurred at:

(12) $${\bar{g}}_{E\to I,h}=1.3,{\bar{g}}_{N\to I,h}=0.13,{\bar{g}}_{I\to E,h}=0.6,\;\gamma_{E\to I,h}=0.01,\gamma_{N\to E,h}=0.01$$

where ‘h’ denotes the parameter value that reflects the state in healthy individuals (Table.2).

We then defined a parameter set that represents the state in SZ by referencing empirical data from prior postmortem studies, which showed percent differences in these parameters ranging from 10% to 30% relative to unaffected comparison subjects (Table.1). To prevent any single alteration from dominating the interaction effect, we uniformly applied a 20% difference to the parameter values for healthy state in order to represent a generalized model for the typical pathological state in SZ. The adjusted parameters for SZ state at 20% difference, as denoted by ‘sz’, were (Table.2):

(13) $${\bar{g}}_{E\to I,sz}=1.04,{\bar{g}}_{N\to I,sz}=0.104,{\bar{g}}_{I\to E,sz}=0.48,\;\gamma_{E\to I,sz}=0.012,\gamma_{N\to E,sz}=0.012$$

Next, we defined a scaling factor λ to systematically adjust the degree of alteration from the healthy to SZ states for each parameter. λ is defined as:

(14) $$\lambda =\frac{x-x_{h}}{x_{sz}-x_{h}}$$

Here, $x_{h}$ and $x_{sz}$ denote parameter values in healthy and SZ states, respectively. By varying λ from 0 to 1, the parameter x can be adjusted from its original value in the healthy state (0% difference where λ=0) to its altered value in the SZ state (20% difference where λ=1). This approach allowed us to methodologically study the effect of synaptic alterations on gamma power across a wide range of parameter values between healthy and SZ states.

Using this approach, we first investigated how varying individual synaptic parameters from healthy to SZ states affects gamma power (Figure.4B). Varying λ from 0 to 1 for ${\overset{‾}{g}}_{E\to I}$ and ${\overset{‾}{g}}_{N\to I}$ simultaneously to model lower E→I synaptic strength resulted in only a 5% decrease in gamma power. Similarly, varying λ for $\gamma_{E\to I}$ and $\gamma_{N\to I}$ simultaneously to model greater variability in E→I synaptic strength resulted in a 3% decrease in gamma power. Finally, varying λ for ${\overset{‾}{g}}_{I\to E}$ to model lower I→E synaptic strength resulted in an 18% deficit in gamma power. Consequently, the additive effect of all these parameter changes was expected to be a 26% reduction in gamma power. However, lower E→I synaptic strength, lower I→E synaptic strength and greater variability in E→I synaptic strength in concert reduced gamma power by 45% (Figure.4B), demonstrating a synergistic interaction among these synaptic parameters in the mean-field model.

To evaluate the robustness of this synergistic effect, we compared gamma power deficits predicted by additive changes with those from the simulated effect across a wide range of percent differences in the synaptic parameters. Our analysis showed that simulating the combined alterations consistently produced greater deficits in gamma power than the additive predictions when the percent difference uniformly applied to all synaptic parameters was in the range of 7.5% to 32.5% (Figure. 4C). Further analysis measuring the fold ratio of gamma power deficits between the simulated effect and the additive prediction showed that the synergy (marked by the fold ratio >1) is the greatest when the parameters differed by 12.5% to 22.5% from their healthy states (Figure.4D). Together, these findings demonstrate that the mean-field model robustly captures the synergistic property of interactions among multiple synaptic alterations found in PFC of SZ.

### 3.5 Two-dimensional bifurcation analyses of synergistic interactions in mean-field model

Finally, we characterized dynamical mechanisms underlying the synergistic interactions of the three SZ-associated alterations via a series of 2D bifurcation analyses of our mean-field model. Specifically, we assessed Hopf bifurcation curve of the two parameters while varying the third parameter from healthy to SZ states as shown in Table 2.

**Fig. 5:**
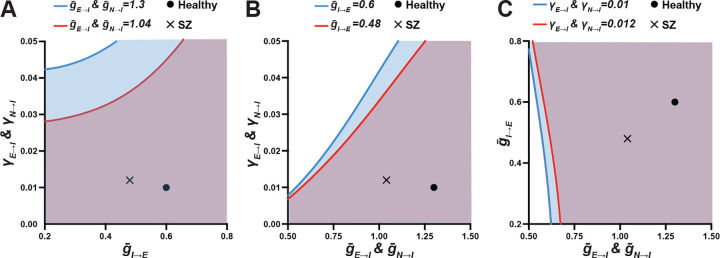
2D bifurcation analyses of the interactions among the synaptic alterations found in PFC of SZ on gamma power. Each bifurcation diagram shows an interaction of two synaptic parameters on the x and y axes while varying the third parameter from its healthy to SZ states as shown on the top of each graph. The colored lines and areas represent the Hopf bifurcation points and the parameter space that support gamma oscillations, respectively, as the third parameter is varied from the healthy (blue) to SZ (red) states. The circles and the crosses indicate the values for the healthy and SZ states, respectively, for the parameters on the x and y axes. The reduction in parameter space that supports gamma oscillations is greatest when the varied parameter is ${\overset{‾}{g}}_{E\to I}$ and ${\overset{‾}{g}}_{N\to I}$ (A), intermediate with ${\overset{‾}{g}}_{I\to E}$ (B), and least with $\gamma_{E\to I}$ and $\gamma_{N\to I}$ (C). Thus, these findings suggest that lower E→I strength is the main synaptic pathology that drives the predicted synergistic effect on PFC gamma power in SZ.

2D bifurcation diagrams of the two synaptic parameters all showed a reduction in the parameter space that supports gamma oscillations when the third parameter was varied from healthy to SZ states (Figure.5). Also, the points that marked the SZ states of the parameters on the x and y axes were placed significantly closer to the bifurcation curve than those that marked the healthy states, further highlighting the reduction in the parameter space that allows the generation of gamma oscillation. Finally, the reduction of this parameter space was the greatest when the varied parameter was E→I strength (Figure.5A), intermediate with I→E strength (Figure.5B), and least with variability in E→I strength (Figure.5C). Together, these observations suggest that although each synaptic alteration can influence the interaction between the other two, lower E→I strength is the main synaptic pathology driving the synergistic interaction and its non-linear effect on gamma power.

## 4 Discussion

In this study, we investigated the macroscopic dynamics of gamma oscillations and their alterations in SZ by deriving a mean-field description of the QIF model network that consisted of all-to-all connected RSEs and FSIs coupled by AMPA, NMDA and GABA synapses. We scaled this QIF model network to the macroscopic level by transforming the membrane potential into a non-dimensional variable and employing Lorentzian distributions for the synaptic strengths and external currents. This approach allowed us to derive a set of firing-rate equations for a network of heterogeneous RSE and FSI populations that are exact in the thermodynamic limit. The firing rate of the mean-field model matched the spiking activity of the finite-size QIF model network for both RSEs and FSIs over a wide range of parameter choices even though the reduction was not exact for the NMDA synapses (see below for a discussion). This close match allowed us to use the mean-field model to reliably explore changes in gamma oscillation dynamics at the macroscopic level.

Prior studies have shown that E→I and I→E synaptic strengths are the critical determinants of cortical gamma power (Sohal, 2022). To investigate how macroscopic dynamics of gamma oscillations are affected by E→I and I→E synaptic strengths in our model, we assessed the effect of ${\overset{‾}{g}}_{E\to I},\;{\overset{‾}{g}}_{N\to I}$ and ${\overset{‾}{g}}_{I\to E}$ on gamma power. Both the numerical simulations and the bifurcation analyses showed that ${\overset{‾}{g}}_{E\to I}$ and ${\overset{‾}{g}}_{I\to E}$ form an inverted-U relationship with gamma power, whereas increasing ${\overset{‾}{g}}_{N\to I}$ reduces gamma power. These findings replicate the effect of each synaptic parameter previously observed in the QIF model network (Rotaru et al., 2011; Chung et al., 2022), validating the utility of our mean-field model in studying the impact of synaptic parameters on the macroscopic dynamics of gamma oscillations.

Mean-field models constructed with Lorentzian distribution assume quenched variability due to the heavy-tailed nature of this distribution. In line with this idea, our mean-field model showed that increasing $\gamma_{E\to I}$ and $\gamma_{N\to I}$ each reduces gamma power monotonically. These findings are also consistent with a prior study that captured the effect of variability in E→I synaptic strength modeled by a Gaussian distribution in a QIF model network (Chung et al., 2022). Thus, the sensitivity of mean-field model to variability provides a computational tool for exploring how increases in synaptic variability might influence gamma oscillation dynamics at the macroscopic level.

A prior study showed that multiple synaptic alterations found in PFC of SZ (i.e., lower E→I strength, lower I→E strength and greater variability in E→I strength) can synergistically reduce gamma power due to the nature of non-linear dynamics of QIF neurons (Chung et al., 2022). This synergistic interaction was also observed in our mean-field model, demonstrating that the non-linearity of synaptic interactions is robustly captured by our model. In addition, the strength of this synergistic interaction was greatest when the magnitude of difference applied to each parameter was comparable to those reported in the postmortem human brain studies of SZ (Table.1). These findings highlight the potential utility of our mean-field model to investigate the dynamic interactions of synaptic alterations modeled by empirically findings in SZ.

The dynamic mechanism by which lower E→I strength, lower I→E strength and greater variability in E→I strength interact to synergistically reduce gamma power in SZ is not well understood. The 2D bifurcation analyses in this study revealed a reduction in the Hopf bifurcation-defined parameter space between two synaptic parameters as the third parameter shifted from healthy to SZ state. Furthermore, the reduction in the parameter space that supports gamma oscillations was most pronounced when the varied parameter was E→I strength. Thus, the shift in Hopf bifurcation due to lower E→I strength could be the primary driver of the synergistic interactions that allow small changes in multiple synaptic parameters to robustly reduce gamma power in PFC of SZ.

Our mean-field model included the dynamics of NMDA synapses, which we modeled as a slow excitatory synaptic current gated by the activity of the AMPA synapses. The dynamics of NMDA synapses typically depend on the pre- and post-synaptic activity, so that an exact mean-field is not technically possible. In low Mg^++^, the dependence on the postsynaptic potential is weak, so that the mean-field approximation is reasonable. Alternatively, recent work (Gast et al., 2021; Chen and Campbell, 2022; Gast et al., 2023) on short-term synaptic plasticity and spike frequency adaptation, all of which are neuron specific, has shown that a mean-field approximation can be justified if there is a dramatic separation of time scales. Since the dynamics of NMDA synapses are markedly slower than the other synaptic processes, the use of the mean-field approximation is reasonable for NMDA synapses. Consistent with this idea, our model showed a close alignment in gamma oscillation dynamics with the QIF model network that included NMDA synapses, validating our approach in modeling the dynamics of these synapses.

In conclusion, we derived a mean-field description of QIF model network to systematically explore the effect of synaptic alterations between RSEs and FSIs and their collective impact on gamma oscillation dynamics in SZ. Our mean-field model robustly captures the macroscopic dynamics of gamma oscillations and their alterations across a wide range of synaptic parameters that are thought to be the key determinants of cortical gamma power. Furthermore, our analyses reveal that the shift in Hopf bifurcation by lower E→I strength could be a critical mechanism by which multiple synaptic alterations reported in PFC of SZ non-linearly interact to synergistically reduce gamma power. As alterations in gamma oscillation dynamics are observed not only in SZ but across a wide spectrum of illnesses including bipolar disorder (Lu et al., 2022), autism (Simon and Wallace, 2016) and Alzheimer’s dementia (Mably and Colgin, 2018), our mean-field model offers a powerful tool to systematically investigate the disease processes of altered neural dynamics in these diverse neuropsychiatric disorders at the macroscopic level.

## Figures and Tables

**Table 1:** Prior postmortem findings in PFC of SZ, their expected effects, and the corresponding parameters in the mean-field model.

Postmortem findings in PFC of SZ	Expected synaptic changes	Relevant parameters in mean-field model
18% fewer excitatory inputs to FSIs (Chung et al., 2016a)	Lower E→I synaptic strength	${\bar{g}}_{E\to I}$ and ${\bar{g}}_{N\to I}$
10–30% lower mRNA and protein levels of GAD67 in FSIs (Glausier and Lewis, 2011; Fish et al., 2021; Hashimoto et al., 2003)	Lower I→E synaptic strength	${\bar{g}}_{I\to E}$
20% and 28% greater variability in protein levels of VGlut1 and PSD95, respectively, in excitatory inputs across FSIs (Chung et al., 2022)	Greater variability in E→I synaptic strength	$\gamma_{E\to I}$ and $\gamma_{N\to I}$

**Table 2:** Comparison between synaptic parameters in mean-field model for healthy and SZ states

Mean-field parameters	Healthy state	SZ state
${\bar{g}}_{E\to I}$ and ${\bar{g}}_{N\to I}$	1.3 and 0.13 (100%)	1.04 and 0.104 (80%)
${\bar{g}}_{I\to E}$	0.6 (100%)	0.48 (80%)
$\gamma_{E\to I}$ and $\gamma_{N\to I}$	0.01 (100%)	0.12 (120%)
